# The Value of Starch-Treated Foods in the Dieto-Therapy of Diabetes-Mellitus

**Published:** 1913-05

**Authors:** 


### Abstracts and Selections.

### The Value of Starch-Treated Foods in the Dieto-Therapy of Diabetes-Mellitus.

"No subject in medicine has, for the past one hundred and fifty years, been given more thought, from a scientific and experimental standpoint, than diabetes, and yet no subject, as to the true pathology and etiology, of which we possess proportionately less accurate information." This is the introductory paragraph of a valuable contribution on the above subject appearing in the March issue of the *Medical Summary*, of Philadelphia, by George Mosse Norton, M. D., Savannah, Ga., who, after considering the pathology, etiology, symptomatology, and prognosis of diabetes-mellitus, says:

"Dieto-therapy offers the greatest and most rational promise of relief or cure, and is by far the sheet anchor in the treatment of diabetes, and is more efficient than any drug or combination of drugs, and no permanent results have ever been obtained without strict dietetic supervision. Unfortunately, pharmacology has not provided any drug which acts directly upon the excitability of the sugar-forming process of the liver. All authorities agree that the diabetic wastes away and starves to death—from consuming his own tissues—through the impaired condition of the 'glycogenic function' of the liver."

He affirms that heretofore the difficulty in the successful management of diabetes has been that the patient could not assimilate foods containing carbohydrates in the form of starch as it appears in the ordinary food products, and by eliminating the starch from the products their value as a sustaining food is completely destroyed.

He also emphasizes the fact—"All the working cells of the body use sugar as their foodstuffs and immediate source of energy, which if not supplied from the food (starch) ingested, must be taken out of the tissues, and in the patient suffering from diabetes the waste from the body is more than the intake of food into the system."

The author's experience coincides with van Noorden and other eminent authorities that the best food for the diabetic is the food containing the greatest amount of carbohydrates which they can tolerate, because in the carbohydrates is contained the greatest proportion of calories, or heat units, which go to make up the energy of life.

Dr. Norton states, that while he realized than van Norden's deductions were correct, yet while entertaining little hope that a starchy food which a diabetic could ingest with impunity would ever be perfected, it was by a mere "coincidence" that his attention was brought to such a food. A prominent New York physician was consulted by one of Dr. Norton's diabetic patients, and was placed on a new starch-treated product known as the Jireh Diabetic Foods.

The doctor, in commenting on the case says: "When his patient returned after three months—all the while eating the starch-treated foods, he was amazed, but agreeably surprised at the remarkable improvement, which continued after the lapse of a year. Health, strength and weight gradually increased on these foods, together with eggs and other suitable diet, and the sugar slowly disappeared from the urine, only traces now being present."

The author refers to the increased death rate from diabetes and avers that "before the present process of refining, bolting, and bleaching flour became common, there were few cases of diabetes in either men or women, but of late years from the constant ingestion of insoluble starchy foods, this disease has increased with leaps and bounds. He points out the amazing fact that the rapid increase in the death rate from diabetes has kept pace with the 'patent roller' process of manufacturing flour."

The doctor in describing the process of treating the starch says, "Each starch granule in cereal food products is inclosed in a tough envelope that the process of grinding does not break. To render these easy of digestion, without the formation of sugar in the diabetic, is the secret of these starch-treated foods."

"The starch granules are thoroughly broken up by diathermous fermentation, produced by the addition of certain digestive enzymes to the flour, which after thorough trituration, is subjected to a certain degree of heat applied for a specified period of time, by especially constructed machinery—designed for this particular purpose."

"The above treatment applied to a whole-wheat-stone-groundflour followed by the scientific application of heat causes a commingling of the carbohydrates and nitrogen molecules of the starch granules of the wheat berry, resulting in a very slight fermentation leading to division and expansion, after ingestion, and to final disintegration in the small intestine."[2]

The author refers to the mineral constituents of the wheat berry as follows: "The wheat berry contains about 75% of starch, and in combination are certain other constituents, gluten, nitrogen, carbon, chlorine, calcium, phosphorus, sulphur, sodium, potassium, ferrum, magnesium, and fluoric acid. Nature placed the above named mineral or cereal salts into the wheat berry that the Biblical injunction might be fulfilled, that bread would really be the 'staff of life' but to change the starch in flour from which the above cereal salts have been eliminated—by present day method of milling—is impossible, and it is likewise impossible to render it soluble so that the dextrin and glucose can be appropriated and oxidized by the various ferments of the digestive tract." Referring to the physiologic and therapeutic value of Jireh Products, the author summarizes as follows:

The foods are manufactured from a whole-wheat-stone-ground-starch-treated flour in which is retained all of the starch and cereal salts so necessary to sustain and build up the depleted system of a diabetic sufferer. The ingestion of these foods will assist in equalizing sugar production to sugar requirements—by enabling the defective function of the diabetic's liver to fix the starch and store the sugar as "glycogen" to be used, as force and energy, as in health.

In closing the author says: "There are ten reasons why foods made from starch-treated flour are superior foods in the dietetic treatment of diabetes.

1. The wheat from which these food products are manufactured is selected from the choicest grade of wheat and ground on the 'old-fashioned' Buhr-millstone.

2. These foods are manufactured from an entire whole-wheat-stone-ground-flour, because the best part of the nutriment is under the shell where the phosphate of potash and other cereal salts—absolutely demanded by the body for its proper sustenance—are found.

3. The flour from which these foods are manufactured is subjected to a diathermous fermentation which produces certain changes in the carbohydrate or starch granules, but retains all the food value of the starch and cereal salts.

4. The change in the starch is slight but sufficient to facilitate its rapid change into a form of sugar to sustain the body in a healthy condition.

5. The foods are palatably delicious and satisfying as compared with the insipid and obnoxious devitalized gluten foods.

6. They contain all the mineral constituents of the whole wheat berry so necessary to maintain the vitaliy of the human body.

7. The starch is not changed into indigestible dextrine or glucose.

8. No chemicals whatsoever are employed in the treatment of the starch.

9. These starch-treated food products are high in food value.

10. They are physiologically correct."

